# Modeling Airflow Using Subject-Specific 4DCT-Based Deformable Volumetric Lung Models

**DOI:** 10.1155/2012/350853

**Published:** 2012-12-20

**Authors:** Olusegun J. Ilegbusi, Zhiliang Li, Behnaz Seyfi, Yugang Min, Sanford Meeks, Patrick Kupelian, Anand P. Santhanam

**Affiliations:** ^1^Department of Mechanical Materials and Aerospace Engineering, University of Central Florida, Orlando, FL 32816, USA; ^2^Department of Radiation Oncology, University of California, Los Angeles, CA 90230, USA; ^3^Department of Radiation Oncology, M.D. Anderson Cancer Center Orlando, Orlando, FL 32806, USA

## Abstract

Lung radiotherapy is greatly benefitted when the tumor motion caused by breathing can be modeled. The aim of this paper is to present the importance of using anisotropic and subject-specific tissue elasticity for simulating the airflow inside the lungs. A computational-fluid-dynamics (CFD) based approach is presented to simulate airflow inside a subject-specific deformable lung for modeling lung tumor motion and the motion of the surrounding tissues during radiotherapy. A flow-structure interaction technique is employed that simultaneously models airflow and lung deformation. The lung is modeled as a poroelastic medium with subject-specific anisotropic poroelastic properties on a geometry, which was reconstructed from four-dimensional computed tomography (4DCT) scan datasets of humans with lung cancer. The results include the 3D anisotropic lung deformation for known airflow pattern inside the lungs. The effects of anisotropy are also presented on both the spatiotemporal volumetric lung displacement and the regional lung hysteresis.

## 1. Introduction

Lung radiotherapy aims at delivering therapeutic ionizing radiation on lung tumor in the form of external beams from different angles while minimizing exposure to surrounding healthy tissues. Errors in the lung tumor localization during therapy may lead to an undertreatment of the tumor and an overexposure of ionizing radiation to the surrounding lung tissues [1]. Lung tumor localization errors occur as the lung deforms during breathing, thereby compromising the accuracy of the radiation therapy [2]. Clinical approach to address these localization errors typically involves increasing tumor margins in radiation treatment plan and avoiding voluntary breathing variations (e.g., sneezing and coughing) [3]. A precise estimation of lung tumor position can be facilitated by a fluid structure interaction model, where the airflow inside the lungs is modeled using computational fluid dynamics (CFD) techniques, and the structure is modeled as a subject-specific anisotropic poroelastic medium. Such an estimation of the lung tumor position will not only lead to improved adaptive radiotherapy and treatment outcomes but also lead to an improved image acquisition guidance in the future. 

CFD of airflow inside lungs during respiration is a challenging task due to the complexity of lung geometry, structural heterogeneity and material anisotropy, and other boundary constraints [4]. Specifically, the human lung is heterogeneous and anisotropic, with a wide range of elastic property values [5]. This situation is further exacerbated by the presence of tumors, which significantly increase the local elastic modulus due to stiffness [6]. Several methods have been used to simulate flow and deformation in the lung, ranging from fractal theory to macroscopic [7]. Some methods allow simulation over several branching levels of the tracheobronchial tree to the alveoli level, but are computationally intensive and impracticable for near real-time application. For instance, Yang et al. [7] documented the computation time for airflow studies in 11 airway branches to be in year's duration. Kunz et al. [8] and Radhakrishnan and Kassinos [9] demonstrated the computational complexity of fluid flow solution by using a parallel CFD solver for studying the convective and diffusive particle depositions inside the lung. The airway branching was modeled up to 11 branches. The rest of the lung space was modeled as a homogenous space. Fluid structure interaction (FSI) between the airflow and the lung parenchymal region was first investigated in [10] by modeling the alveolar region as macro-air-sacs with isotropic elastic properties. Coupling of anisotropic elastic properties for lung substructures with CFD studies has not been previously investigated. 

This paper describes a methodology for coupling the anisotropic elasticity with CFD analysis to effectively predict the volumetric lung displacement at different breathing phases and, by so doing, track tumor motion. The fluid structure interaction depends critically on the anisotropic nature of the subject-specific lung tissue elasticity. To incorporate the anisotropic tissue elasticity, a multizone-based geometric representation is employed. While multizone representations have been previously investigated for arterial blood flows [12], it has not been used for investigating airflow inside the lungs. The usage of such a geometric representation avoided errors in airflow analyses caused by airway segmentation errors and reduced the computation time as compared to the timings previously reported in [7]. The airflow modeling studies also demonstrated the influence of anisotropic elasticity estimated from 4DCT on the resulting airflow inside lungs and the volumetric deformation. The combined usage of anisotropic elasticity and a multizone geometry representation form the key contribution of the paper.

## 2. Formulation

The present study considers the lung as an anisotropic poroelastic medium. The spatially varying Young's modulus (YM) data is adopted from those derived based on optical flow registration of human data in a previous study [13]. The mathematical model involves simultaneous solution of the equations governing fluid flow of air through the airway and the structural deformation of the lobe. A gauge pressure acquired from the patient using spirometry is imposed at the trachea, which in turn drives air into the lobes, and the pressure of the air inside the lobe in turn results in lung deformation during the breathing process. This flow-structure interaction (FSI) approach enables the prediction of the spatial velocity distribution and lung displacement over several breathing cycles. In order to investigate the impact of nonlinear elastic property, the predicted deformation with and without allowance for spatial variation in the YM is compared. The FSI equations were solved by means of ADINA commercial computational code [14].

### 2.1. Estimating Tissue Elastic Properties from 4DCT Images

The 4DCT scans used in this study were acquired at the M.D. Anderson Cancer Research Center, Orlando, from *in vivo *experiments on human adult patients at different times of the breathing cycle. 4DCT datasets for a human subject at 10% tidal volume intervals were taken using Siemens Biograph strain-gauge 64 slices CT. The 3D volumetric lung and the airways were segmented using Pinnacle MBS and OSIRIX software.

The 4DCT data registration algorithm was used to estimate the motion of each 3D voxel at the end-expiration 3D volume data by searching for and locating a corresponding voxel in another 3D volume at a different breathing phase. An optical flow-based motion estimation, which is based on local Taylor series approximation and is further described in the optical flow literature, was used for the registration [15]. One of the limitations of the optical flow method as applied to estimating the 3D organ motion was the low sensitivity to variations in regional motion. In order to improve the accuracy of optical flow algorithm implementation, we used a multilevel, multiresolution optical flow method [16], which computed optical flow between two 3D volumes at lower resolution, propagated the result to the higher resolution volume, and subsequently to the original resolution volume data. In this approach, the organ anatomy was separated into four parts: (1) lung outline, (2) large capillaries, (3) small capillaries, and (4) parenchymal region. At each level of anatomy optical flow, a multilevel, multiresolution optical flow registration was used for computing the 4D organ motion of that anatomy and integrated into the next level. 

The next step was to estimate the subject-specific deformation model's kernel for the surface and the volumetric lung representations, which represents the internodal elastic interaction, and the surface lung elasticity in terms of the YM values. The method is based on the approach discussed in [17]. The volumetric lung deformation operator took as input the force applied on the voxels inside the lung and computed the subsequent change in shape. We first estimated the volumetric applied force and the displacement, which were the inputs for estimating the operator. The force applied on a lung for a given change in volume was computed using the pressure-volume curve measurement, a key pulmonary function test. This force was then spatially distributed inside the lung using the vertical gradient of pressure. Such a distribution estimated the volumetric applied force. 

The volumetric lung displacement was estimated using the optical flow, with Euclidean distance-based interpolation of surface registration. We then estimated the surface lung deformation operator as previously discussed in [16]. For both the surface and volumetric lung deformation, a heterogeneous Green's function (GF) based formulation was considered. The structural and functional constants estimated for the surface lung dynamics were specifically used for the volumetric lung dynamics. The GF for the volumetric lung was reformulated in the spectral domain using a hyper spherical harmonic (HSH) transformation. Upon simplification, the HSH coefficients of the displacement were represented as a product of the HSH coefficients of the applied force and the deformation operator. The formulation at this stage was mathematically ill-posed since the dimension of the HSH coefficients was higher than the volumetric displacement and the applied force. Local isometricity was assumed at each volumetric point obtained using the *structural* and *functional constants* associated with each voxel. The constraints were computed from the values associated with the lung surface points using a surface lung deformation model [18]. This approach reduced the dimensionality of the deformation operator, making the formulation well-posed. Thus for known values of the applied force and displacement, the HSH coefficients of the operator were estimated, and the YM value of each volumetric point was computed.

### 2.2. Formulation of Mathematical Model for Computational Fluid Dynamics Simulation

The mathematical model involves solution of the coupled poroelastic flow-structure interaction equation with nonhomogeneous and anisotropic tissue properties. This coupled field approach required the solution of the Richard's equation [19] for the local lung pressure and velocity distributions, given by

(1) $$\begin{array}{c} \emptyset \beta \frac{\partial p}{\partial t}=\nabla \times \left[\frac{k}{\mu }\left(\nabla p+\rho g\right)\right]-\frac{\partial }{\partial t}\left(\frac{\partial u}{\partial x}+\frac{\partial v}{\partial y}+\frac{\partial w}{\partial z}\right), \end{array}$$

where *∅* and *k* represent the porosity and permeability of the tissue, respectively, *β* and *μ* represent the compressibility and viscosity of air, respectively, *p* is the local pressure (pore pressure), *ρ* is air density, and *u*, *v*, and *w* are the three components of the deflection (deformation) vector for the tissue. Note that the above equation for pore pressure has already incorporated the Darcy equation for gas flow through the tissue skeleton. This equation was coupled to the lung elastic deformation by the presence of the dilatation (final term) in the above equation. This term was supplied by solving the elastic deformation field, *u*, from the following poroelastic version of the Navier's equation:

(2) G∇2u→+G1−2v∇(∇×u→)=∇p−f→,

where *G* and *v* are the tissue Shear Modulus and Poisson ratio, respectively, and *f* is an external body force term that can include thermal effects as desired. Note that in (2) *G* represents anisotropic shear modulus. Assuming orthogonal anisotropy the value of *G* is allowed to vary in the *xy*, *yz*, and *xz* planes. The shear modulus is related to the YM through the standard relation (*G* = YM/[2(1 + *v*)]) in each direction. Together the previous two equations provide the full description of the coupled lung flow problem. Solution of these equations is accomplished for subject-specific patient lung geometries using ADINA computational code [14].

### 2.3. Geometry Reconstruction and Multilayer Mesh Generation

**Figure 1:**
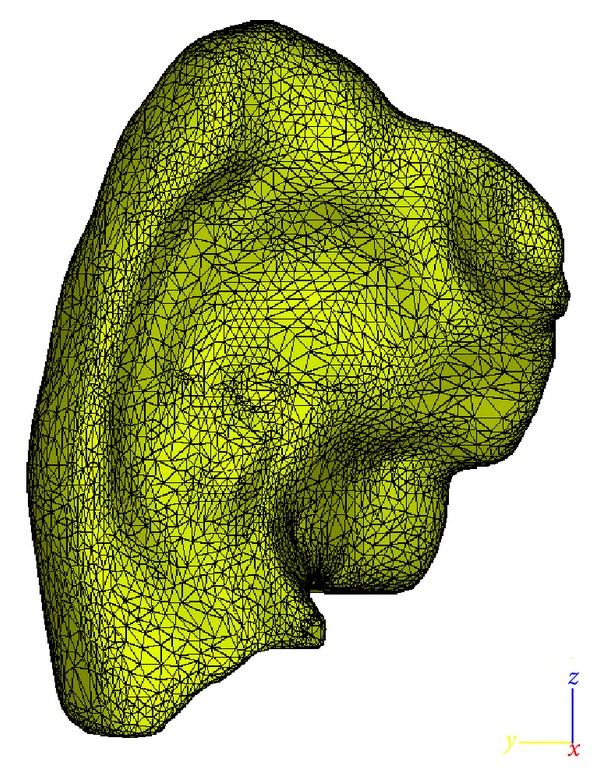
The right lung reconstructed from 4DCT scan dataset obtained at M.D. Anderson Cancer Center, Orlando.

The CT scans at the end-expiration stage are first segmented and used to generate the three-dimensional (3D) geometry utilizing the Mimics computer code [20]. The 3D meshes obtained are then remeshed by means of the 3-matic framework [21] for numerical computation. The resulting geometry reconstructed for the right lung is shown in Figure 1. 

**Figure 2:**
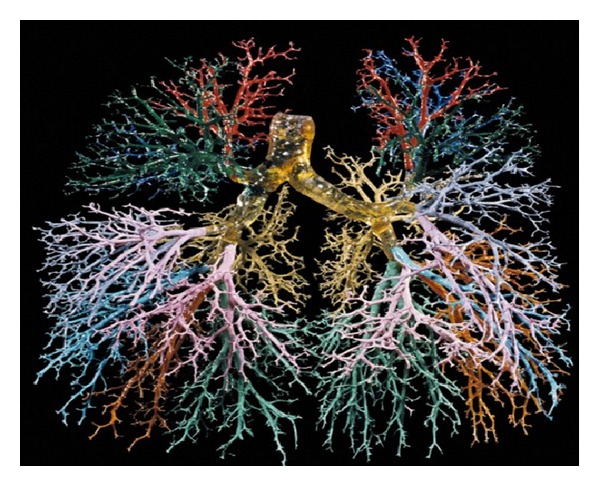
Multilevel branching structure of the human lung [11].

**Figure 3:**
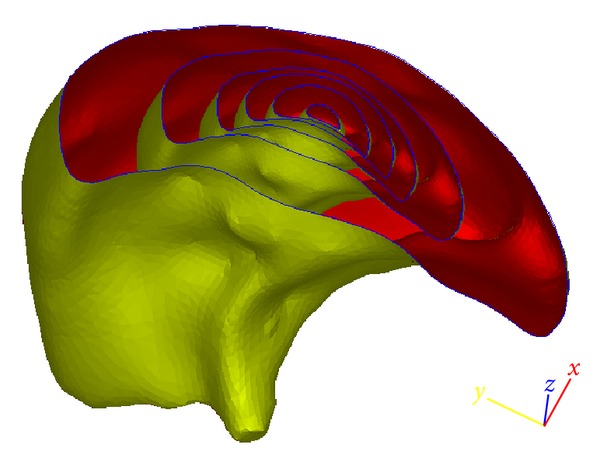
Section view of the multilayer grid structure.

**Figure 4:**
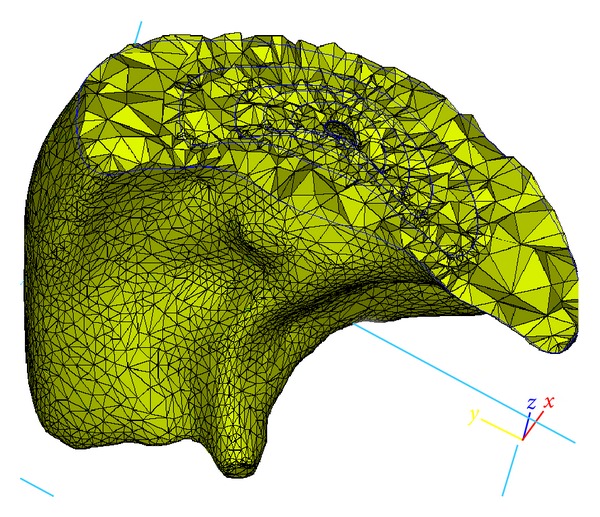
Volume mesh for the multilayer structure.

The lung airway is like a multilevel branching tree as shown in Figure 2 [11]. Based on this airway structure of the lung, the permeability (*K* = *φR* ^2^/8, in which *R* is the branch radius) should decrease significantly from the main central branches to the tip branches as the branch diameter progressively decreases. Correspondingly, the airflow velocity in the primary central branches is significantly higher than that in the peripheral branches. A relatively fine volume numerical mesh size will therefore be required in the core region to reflect the relatively higher pressure, velocity, and stress gradient there compared to the outer layers. Since proper representation of the grid structure is critical to numerical stability, the multibranching lung structure is approximated within the context of the poroelastic model used here, as a multizone structure shown in Figure 3, that permits application of different grids and, if needed, different property values. It can be seen that the multizone geometry representation is similar to the hyperspherical formulation used for representing the YM values of lung substructures. For instance, a normalized airway branch radius can be converted to permeability values associated with each voxel. This permeability is then allowed to vary from the core (inner shell) to the peripheral layers (outer shell) using the same hyperspherical parameterization. The sectional view of the volume mesh generated is shown in Figure 4. It should be noted that both Figures 3 and 4 represent cut-out views of the lobe in order to visualize the multi zone structure and the numerical grid employed.

### 2.4. Input and Boundary Conditions

**Figure 5:**
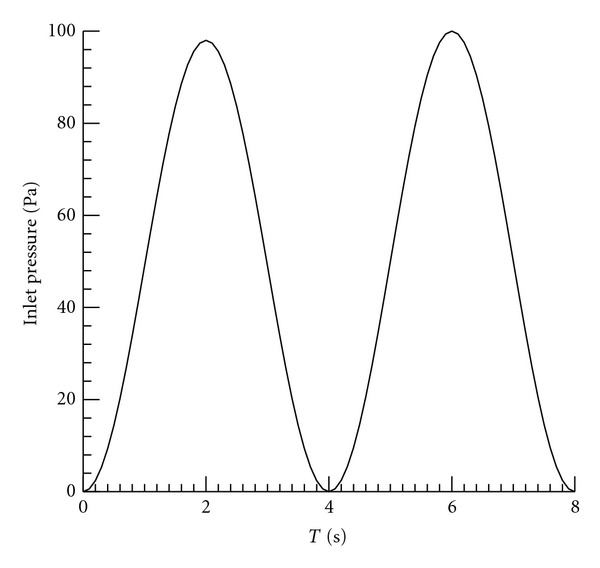
Prescribed inlet pressure.

**Figure 6:**
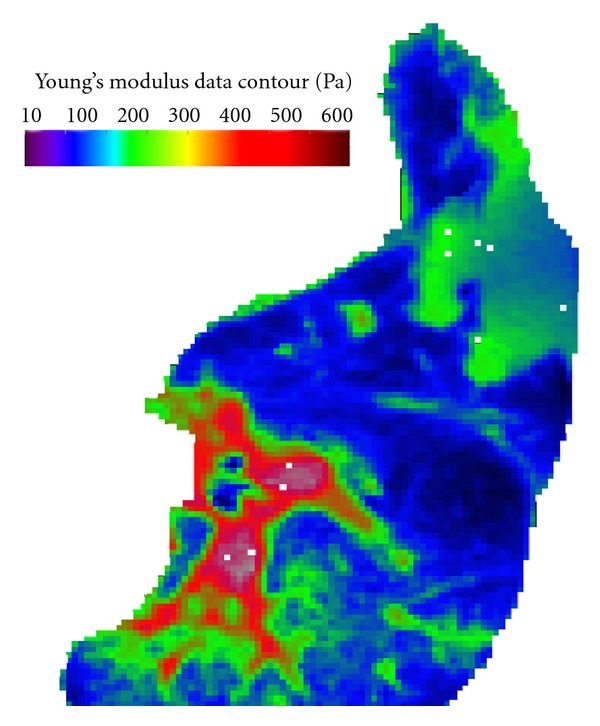
Anisotropic YM obtained based on 4DCT scan dataset and measured pressure-volume curves for patients with lung cancer.

Phasic pressure with a period of 4 s is imposed at the inlet to the lobe as illustrated in Figure 5. The amplitude of the pressure waveform was prescribed based on spirometry studies acquired from M.D. Anderson Cancer Center, Orlando. The property data (beside the YM) were assumed from those established in previous studies. The Poisson ratio *v* in the poroelastic governing equation was assumed to be 0.4, which falls within the range (0.25–0.47) suggested in previous studies [22, 23]. The density of lung was assumed to be 700 kg/m^3^ [21]. Preliminary studies indicated that the deformation is little affected by the permeability *k* over the range (0.01–0.1) as expected. The anisotropic YM adopted from a previous study based on optical flow registration patient data ranged from 10 Pa to 500 Pa. The high YM values correspond to either the tumor location where the structure is rigid or the main trachea wall where the tissue is thick and rigid. The average YM for the whole lung is 178 Pa. This average value is used for the reference cases utilizing linear elastic property.  Figure 6 shows representative color-coded YM distribution on a 2D slice of lobe obtained from optical flow registration and used for the anisotropic elasticity calculations in this paper [13].

## 3. Results

**Figure 7:**
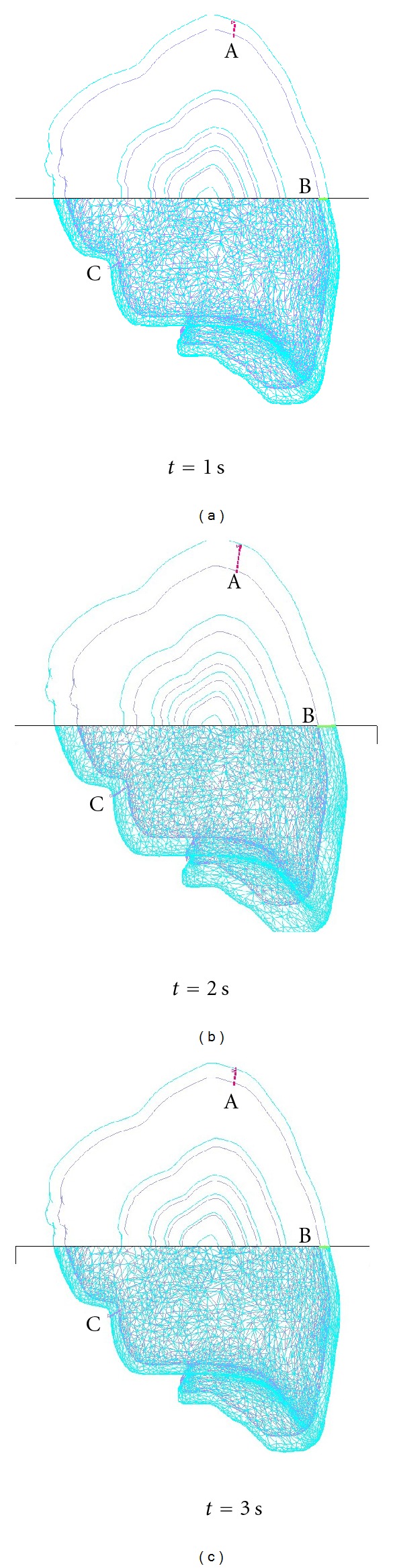
Predicted lung deformation with linear YM.

**Figure 8:**
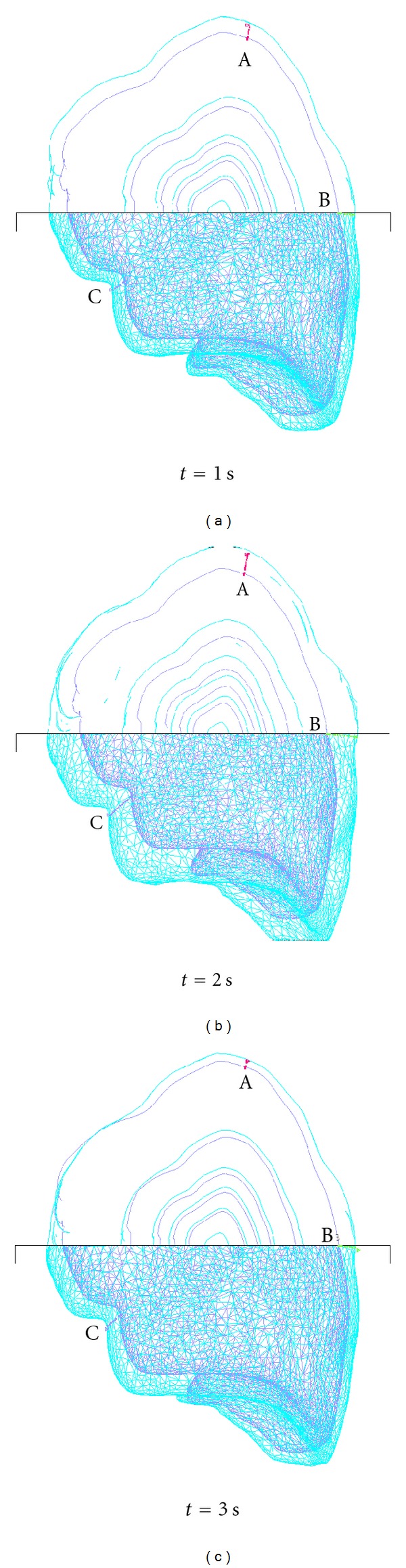
Predicted lung deformation with anisotropic YM.

The predicted lung deformation simulated using the flow structure interaction at different breathing phases is plotted in Figures 7 and 8 for the linear and anisotropic YM cases, respectively. The upper part of each figure is a cut-off view to illustrate the evolution of selected layers from the initial state over the specified duration.  Figure 7 shows that with linear elasticity, the layers expanded monotonically in all directions. On the other hand, Figure 8 shows that the case with anisotropic elasticity exhibits both directional deformation as well as expansion. 

Three nodes or landmarks on the lung surface, marked A, B, C in Figures 7 and 8, were monitored, and their displacements along the *x*,*y*, and *z* coordinate directions were analyzed. Landmark A was at the top surface of the lobe, B was at the outer surface near the rib cage and close to the midpoint along the craniocaudal axis, and C was at the interior surface of the lobe.

**Figure 9:**
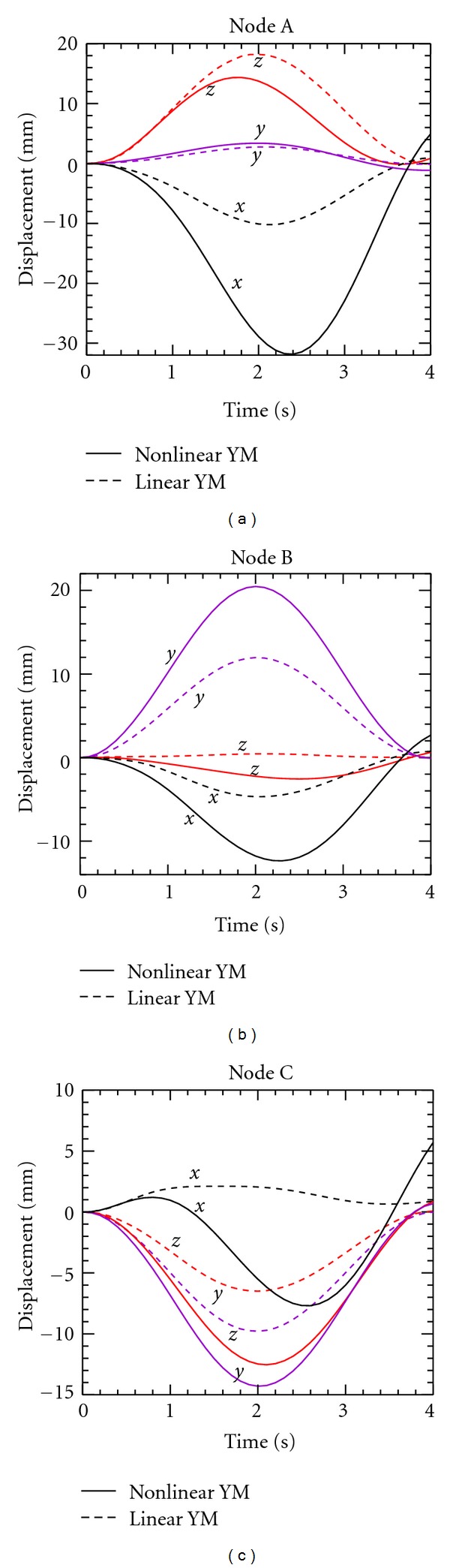
Predicted displacements for monitored nodes A, B, and C for linear and anisotropic YM over the first respiration cycle.

Figure 9 shows the *x*, *y*, *z* displacements of the monitored nodes A, B, and C over the first respiration cycle for both linear and anisotropic elasticity. The predicted displacements with linear and anisotropic YM are quite distinct. The displacement profiles for the linear YM case, which are represented by the dash lines, are generally sinusoidal with a symmetric axis at *t* = 2 s, corresponding to the sinusoidal pressure waveform imposed at the inlet to the lobe. On the other hand, the displacement trajectories for the anisotropic YM case, which are represented by the solid lines in each figure, are distorted from the sinusoidal pressure condition. The peak *x* displacement for node A is located at *t* = 2.4 s, which lags the peak inlet pressure at *t* = 2.0 s by 0.4 s. The peak *z* displacement for node A is 0.2 s ahead of the peak inlet pressure. A similar hysteresis phenomenon is observed for the anisotropic YM case at nodes B and C. In addition, the peak displacements in the *x* direction for nodes A and B in the anisotropic YM model are nearly 3 times the displacements in the linear YM model. These results clearly show that the effect on deformation of anisotropic lung elasticity could be significant.

**Figure 10:**
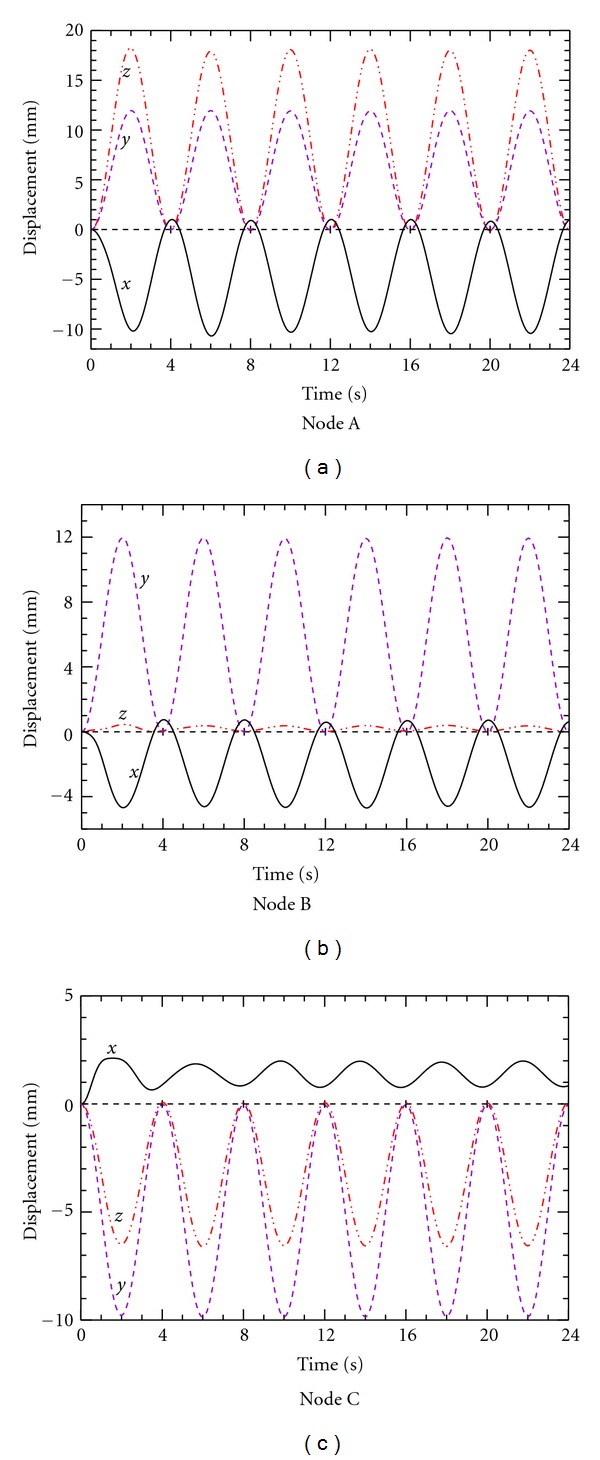
Predicted displacement for nodes A, B, and C over 6 breathing cycles with isotropic YM.

In order to further examine the effect of anisotropicity, the calculations were continued for additional breathing cycles.  Figure 10 shows the *x*, *y*, *z* displacements of nodes A, B, and C for linear YM over 6 breathing cycles. The entire displacement wave pattern becomes stable after the second breathing cycle. The result indicates that all the peak displacement values occur at the midpoint of each cycle, that is, at *t* = 2 s, 6 s and 10 s. The displacements at the end of each cycle are nearly negligible. It is worth noting that in the consensus of the result presented in a previous Figure 9, the displacement profile observed with linear elasticity follows closely the input pressure wave pattern.

**Figure 11:**
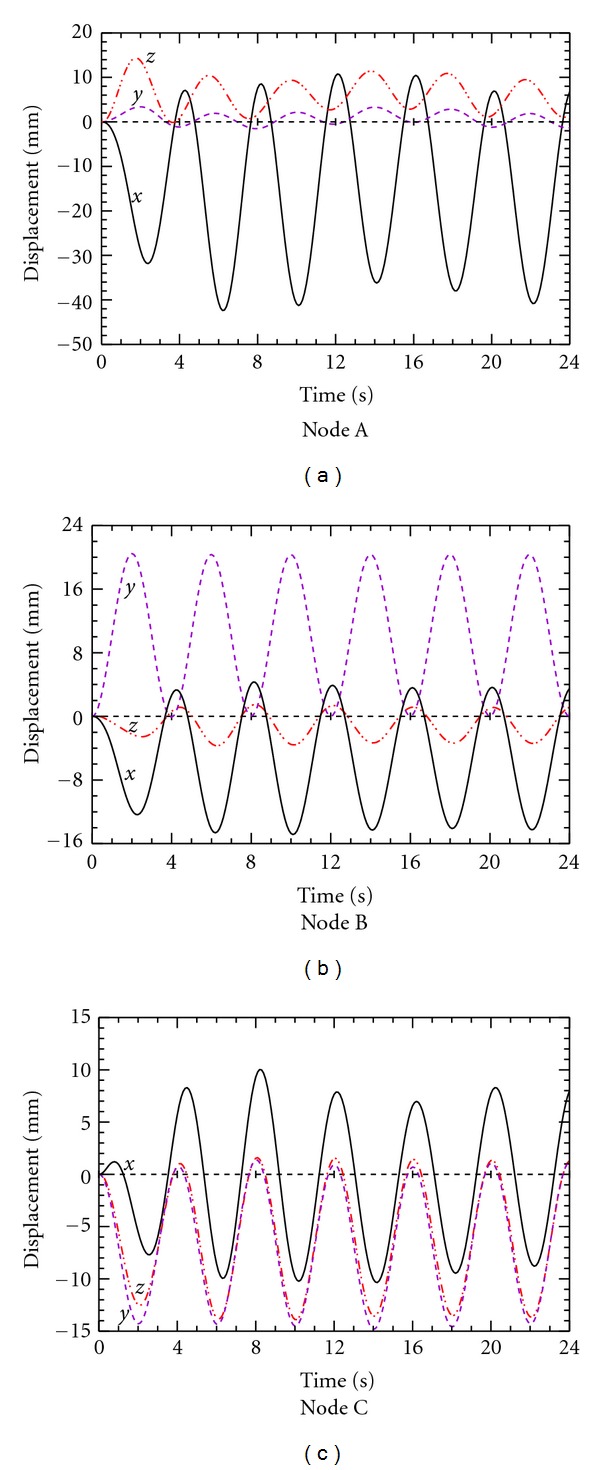
Predicted displacement for nodes A, B, and C over 6 breathing cycles with anisotropic YM model.

The corresponding results over 6 breathing cycles utilizing anisotropic elasticity are presented in Figure 11. The observed hysteresis time for the peak wave appears to be a fixed value for each monitored location. For example, the predicted peak wave of the *x* displacement lags the peak pressure inlet by 0.4 s, 0.3 s, and 0.2 s for nodes A, B, and C, respectively. Note that the peak displacements also vary from cycle to cycle. The observed hysteresis resulted from the anisotropic elasticity distribution in the lung. The hysteresis time is also found to be dependent on the geometric location of the monitored point in the lobe.

**Figure 12:**
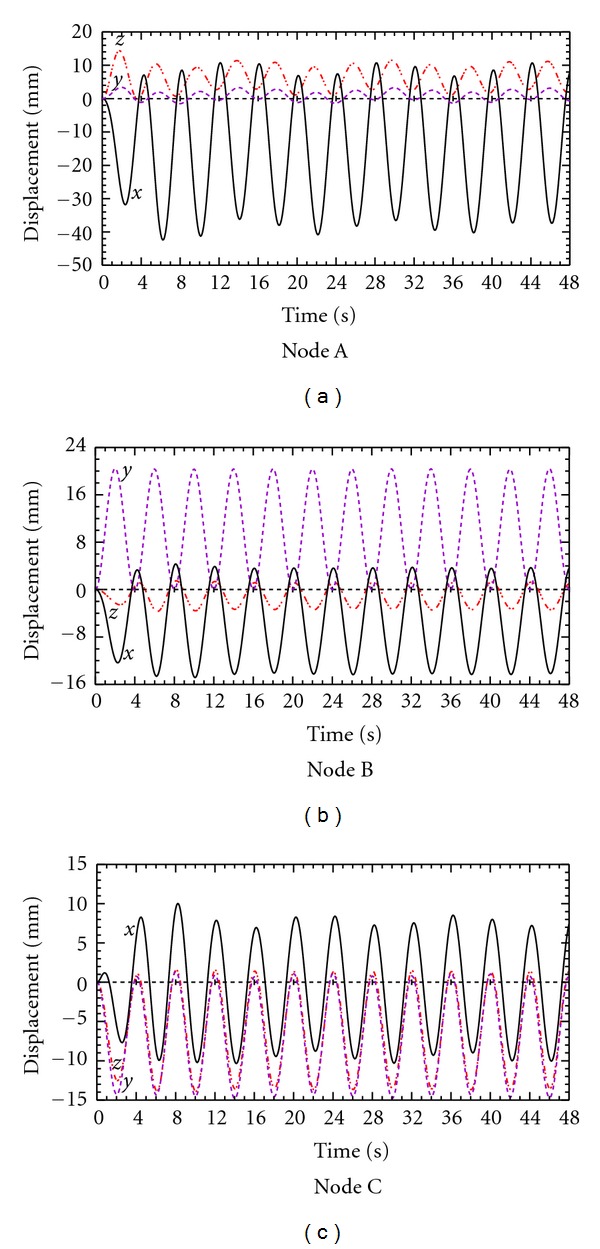
Predicted displacement for nodes A, B, and C over 12 breathing cycles with anisotropic YM model.

In order to further examine the peak variation in the anisotropic case, the calculations are extended to 12 respiration cycles, and the results are plotted in Figure 12. The results indicate that the *x* displacement magnitude profile for the monitored node A reaches local peaks at the 2nd, 6th, and 10th cycles, corresponding to a period of 16 s. The *y* and *z* displacement profiles reach their local maxima at the 4th, 8th, and 12th cycles, which also correspond to a period of 16 s. A similar periodic pattern is observed for node C while the trend in node B is not quite so distinct, perhaps because the latter was located near the symmetry point along the craniocaudal axis. 

**Figure 13:**
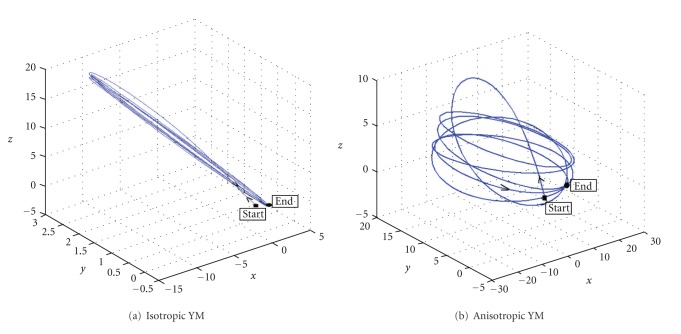
Predicted trajectories of monitored node A over 6 breathing cycles with (a) isotropic and (b) anisotropic YM.

Figure 13 summarizes the results presented above by tracing the trajectories of monitored point A over 6 breathing cycles with both linear (Figure 13(a)) and anisotropic elasticity (Figure 13(b)). The start and end locations of the node are indicated in each figure. Hysteresis is clearly evident in the anisotropic result as exemplified by the significant differences in the trajectories of the monitored point over successive breathing cycles. The trajectories for the linear case on the other hand are nearly coincident. The results for nodes B and C exhibit similar trends and are not presented here for brevity. 

Validation of airflow modeling is essential in verifying the usage of anisotropic YM values. Studies were conducted to verify the accuracy of the lung deformation using the CFD-based flow analysis. Validation consisted of two parts, namely, numerical accuracy and comparison with data. Numerical accuracy was first tested by repeating the calculations for a set of grid numbers below and above the ones chosen for the above results. The results were found to be essentially independent of grid number (and, correspondingly, grid size) beyond the ones chosen for the results. Next, clinical experts delineated two sets of 20 landmarks on two lung models. The motion of the landmarks during the CFD simulation was documented and compared with the displacement observed in the 4DCT dataset that was used to generate the 3D geometry.  Table 1 tabulates the mean target deformation error (TDE) for the displacement obtained using the isotropic and anisotropic YM values. It can be seen that a better accuracy was obtained for the anisotropic case as compared to the isotropic case. The maximum error for both the validations was with 3 mm for the anisotropic case, which is within the clinically acceptable accuracy range. This validation shows that using anisotropic YM values for modeling airflow inside lungs and the subsequent fluid structure interaction can be achieved within clinically acceptable accuracy range using anisotropic elastic values. 

## 4. Conclusion

The effect has been investigated of using subject-specific anisotropic lung elasticity for studying the airflow-induced lung deformation during radiotherapy. The lung was modeled as an anisotropic poroelastic medium. The lung geometry at the end-expiration was reconstructed from 4DCT dataset of patients with non-small-cell lung cancer (NSCLC). The subject-specific tissue elasticity was obtained from the 4DCT using an inverse deformation analysis [10]. The airflow-tissue interaction model involved solving the coupled equations governing fluid dynamics of airflow inside the lungs and the associated structural deformation of the lung, subject to appropriate boundary conditions. 

The major findings of the study may be summarized as follows.

- The local anisotropy in the elasticity of the lung substructures has a significant impact on the airflow inside lungs.
- Anisotropic YM of lung substructures produces a hysteresis effect on the predicted spatial lung displacement relative to the pressure waveform imposed at the inlet to the lung.
- The hysteresis time spatially varies from one 3D location to another inside the lungs.

The above findings have profound implication in the optimization and targeting of radiation to tumor in radiotherapy. The presence of tumor (focal or distributed) in the lung substructures may alter the anisotropism in the lung poroelasticity and, hence, as this study has indicated, significantly affects the resulting spatiotemporal displacement and deformation of the lung. In addition, changes in the tumor regression may lead to changes in the overall anisotropism of the tissue elasticity and subsequently the hysteresis. The net result is an evolving tumor location that is significantly distinct in shape from one breathing signal to another. The model presented here has demonstrated the capacity to fully represent and quantify such detailed motion of any location in the lung utilizing subject-specific tissue elasticity for lung substructures.

Computation time is key limitation for many CFD-based analyses. The proposed method of geometry representation took approximately 24 hours to finish the breathing simulation, which is improvement to previous run time calculations using a full airway geometry. Future work would focus on using high performance graphics units to accelerate the calculations so that the process can be finished in much lesser timeframe.

Results section discusses validation results for the fluid structure interaction, which showed that anisotropic YM values were effective in modeling the airflow inside the lungs. Validating the airflow inside the lungs for different breathing patterns and the subsequent interaction studies will be a key part of our future work. Such a study would involve the use of tracking landmarks in the lung anatomy by means of 2D cine MRI imaging that can acquire the 2D lung snapshots in a given plane in real-time. In addition, using 4D gated MRI imaging, we can validate the volumetric lung deformation and the fluid structure interaction studies taking into account physiological factors such as tumor regression and day-to-day breathing changes. Future studies would also account for the effect of cardiac motion on lung imaging. Such cardiac motion can be acquired using 2D cine MRI imaging in addition to 4D imaging acquired for treatment purposes and modeling the lung deformation subject to being constrained by the cardiac motion. 

## Figures and Tables

**Table 1:** The mean target deformation error (TDE) for the displacement.

4DCT breathing phase	Validation dataset 1		Validation dataset 2	
	Isotropic case (mm)	Anisotropic case (mm)	Isotropic case (mm)	Anisotropic case (mm)
10%	4.11	2.43	4.55	2.61
20%	**4.21**	2.44	**4.43**	3.10
30%	4.13	2.42	4.67	3.04
40%	4.38	2.57	4.81	2.57
50%	4.37	2.29	4.51	2.89
60%	4.74	2.44	4.80	2.02
70%	4.49	2.42	5.23	2.70
80%	5.43	2.17	5.31	2.98
90%	5.47	2.38	5.15	2.10
100%	5.34	2.57	5.40	2.90

## References

[1] Keall PJ, Mageras GS, Balter JM (2006). The management of respiratory motion in radiation oncology report of AAPM Task Group 76. *Medical Physics*.

[2] Santhanam AP, Willoughby T, Shah A, Meeks S, Rolland JP, Kupelian P Real-time simulation of 4D lung tumor radiotherapy using a breathing model.

[3] Guckenberger M, Richter A, Boda-Heggermann J, Lohr F (2012). Motion compensation in radiotherapy. *Critical Reviews in Biomedical Engineering *.

[4] Anderson J *Computational Fluid Dynamics—The Basics with Applications*.

[5] Santhanam AP, Hamza-Lup FG, Rolland JP (2007). Simulating 3-D lung dynamics using a programmable graphics processing unit. *IEEE Transactions on Information Technology in Biomedicine*.

[6] Ioncica AM, Malos A, Crisan E, Popescu C, Saftoiu A, Ciurea T (2010). State-of-the art endoscopic imaging in lung cancer: should specialties collide or concur?. *Journal of Gastrointestinal and Liver Diseases*.

[7] Yang XL, Liu Y, Luo HY (2006). Respiratory flow in obstructed airways. *Journal of Biomechanics*.

[8] Kunz RF, Haworth DC, Porzio DP, Kriete A Progress towards a medical image through CFD analysis toolkit for respiratory function assessment on a clinical time scale.

[9] Radhakrishnan H, Kassinos S CFD modeling of turbulent flow and particle deposition in human lungs.

[10] Ding H, JiangY, Furmanczyk M, Prekwas A, Reinhardt LM Simulation of human lung respiration process using 3-D CFD with macro air sac system model.

[11] Villard PF, Beuve M, Shariat B, Baudet V, Jaillet F Simulation of lung behaviour with finite elements: influence of bio-mechanical parameters.

[12] Ilegbusi OJ, Velaski-Tuema E A fluid-structure interaction index of coronary plaque rupture.

[13] Santhanam AP, Neelakkantan H, Min Y (2011). Visualization of 3D volumetric lung dynamics for real-time external beam lung radiotherapy. *Studies in Health Technology and Informatics*.

[15] Vaina LM, Beardsley SA, Rushton SK (2004). *Optical Flow and Beyond*.

[16] Santhanam AP, Min Y, Rolland JP, Imielinska C, Kupelian P (2011). *4DCT Lung Registration Methods*.

[17] Santhanam AP, Min Y, Mudur SP (2010). An inverse hyper-spherical harmonics-based formulation for reconstructing 3D volumetric lung deformations. *Comptes Rendus-Mecanique*.

[18] Santhanam A, Mudur S, Rolland J (2006). *An Inverse 3D Lung Deformation Analysis for Medical Visualization*.

[19] Richards LA (1931). Capillary conduction of liquids through porous mediums. *Journal of Applied Physics*.

[20] http://www.materialise.com/mimics.

[21] http://www.materialise.com/BiomedicalRnD/3-matic.

[22] Werner R, Ehrhardt J, Schmidt R, Handels H Modeling respiratory lung motion—a biophysical approach using finite element methods.

[23] Chhatkuli S, Koshizuka S, Uesaka M (2009). Dynamic tracking of lung deformation during breathing by using particle method. *Modelling and Simulation in Engineering*.

